# Enhancing Microservice Security Through Vulnerability-Driven Trust in the Service Mesh Architecture

**DOI:** 10.3390/s25030914

**Published:** 2025-02-03

**Authors:** Rami Alboqmi, Rose F. Gamble

**Affiliations:** Department of Computer Science, Tandy School of Computer Science, University of Tulsa, Tulsa, OK 74104, USA; gamble@utulsa.edu

**Keywords:** telecommunication, B5G, 6G, zero trust, cloud-native, vulnerability scanning, service mesh, trust management, container security, threat sharing, automation

## Abstract

Cloud-native computing enhances the deployment of microservice architecture (MSA) applications by improving scalability and resilience, particularly in Beyond 5G (B5G) environments such as Sixth-Generation (6G) networks. This is achieved through the ability to replace traditional hardware dependencies with software-defined solutions. While service meshes enable secure communication for deployed MSAs, they struggle to identify vulnerabilities inherent to microservices. The reliance on third-party libraries and modules, essential for MSAs, introduces significant supply chain security risks. Implementing a zero-trust approach for MSAs requires robust mechanisms to continuously verify and monitor the software supply chain of deployed microservices. However, existing service mesh solutions lack runtime trust evaluation capabilities for continuous vulnerability assessment of third-party libraries and modules. This paper introduces a mechanism for continuous runtime trust evaluation of microservices, integrating vulnerability assessments within a service mesh to enhance the deployed MSA application. The proposed approach dynamically assigns trust scores to deployed microservices, rewarding secure practices such as timely vulnerability patching. It also enables the sharing of assessment results, enhancing mitigation strategies across the deployed MSA application. The mechanism is evaluated using the Train Ticket MSA, a complex open-source benchmark MSA application deployed with Docker containers, orchestrated using Kubernetes, and integrated with the Istio service mesh. Results demonstrate that the enhanced service mesh effectively supports dynamic trust evaluation based on the vulnerability posture of deployed microservices, significantly improving MSA security and paving the way for future self-adaptive solutions.

## 1. Introduction

Cloud-native technologies revolutionize cloud computing, enabling scalable and resilient microservice architecture (MSA) applications [1]. The rising adoption of MSAs [2] is driven by sectors like telecommunications [3], finance [4], and healthcare [5], seeking agile, robust architectures for evolving demands. Mordor Intelligence [6] forecasts the MSA market to grow from USD 1.63 billion in 2024 to USD 4.57 billion by 2029. This rapid growth highlights the pivotal role of MSAs in modern software development and their increasing importance in driving digital transformation, especially in sectors such as telecommunication. In the telecommunication industry, future networks, such as Fifth- and Sixth-Generation (5G/6G) networks—collectively referred to as Beyond 5G (B5G)—rely heavily on MSAs to create dynamic networks that adapt to evolving business needs. In these networks, core hardware is built as software-defined, removing hardware dependencies and enhancing flexibility.

The MSA’s transformative potential encounters significant challenges, notably the necessity for ongoing monitoring of microservices [7]. MSA applications demand strong mechanisms for securing sensitive data through identification, authentication, authorization, and accounting security measures. A microservice breach can lead to widespread compromise of the entire MSA application. Effective security measures, including access control policies, are essential [8] given the synchronous and asynchronous communication of microservices [9]. In cloud-native environments, within 6G networks, orchestration and service mesh solutions are vital for addressing varied security needs, including findings of microservice trustworthiness. Although Taleghani et al. [10] provide a comprehensive survey of trust evaluation techniques in 6G networks, including four works focused on cloud computing, these studies do not explicitly address evaluating the trustworthiness of deployed microservices using service mesh-based approaches. According to [11], the service mesh plays a crucial role in B5G networks, such as 6G by offering functionalities that include microservice discovery, observability, load balancing, security, and management.

Introduced in 2017, the service mesh comprises a control plane for managing operations and a data plane for secure microservice communication via sidecar proxies [12]. While still in early adoption, it has gaps in dynamic microservice management, real-time policy enforcement, and self-adaptive security solutions [13]. Current implementations address basic needs but can evolve to include adaptive controls such as data labeling and encryption based on a vulnerability assessment of each deployed microservice. Key advantages of using a service mesh include intercepting all microservice communications [14] and using sidecars as policy enforcement points [15]. The pivotal role of the service mesh in post-B5G networks is highlighted as a critical solution for security and management challenges [11,16].

Deploying MSA applications often relies on containerization, a lightweight virtualization technique that packages a microservice and its dependencies in an isolated environment. This approach enhances portability and scalability, allowing microservices to replicate as needed to meet dynamic demands. However, increased scalability also expands the attack surface as the number of deployed microservices grows. Orchestration solutions like Kubernetes [17] efficiently manage large-scale container deployments but lack robust mechanisms to detect malicious microservices during runtime. Integrating a service mesh as an infrastructure layer within Kubernetes can improve security by enabling mutual authentication and encrypted microservice-to-microservice communication. However, a service mesh may fail to detect compromised microservices, especially those operating under full implicit trust [18,19]. This implicit trust can allow attackers to exploit compromised microservices to infiltrate and potentially breach the entire MSA application [20].

Compromised microservices can lead to significant threats, including the loss of privileged access [21], address resolution protocol spoofing, distributed denial-of-service attacks, sniffing, and tampering [22,23]. The Apache Log4j vulnerability in 2021, for instance, enabled remote code execution [24], highlighting the potential consequences of inadequate runtime protections for MSA applications. MSA applications are particularly vulnerable, with an average of 180 vulnerabilities per MSA application compared to 39 in a monolithic architecture application [25]. These vulnerabilities expose microservices to various risks, including privilege escalation and unauthorized access [26]. High-profile incidents, such as the exploitation of over 50,000 vulnerable containers for unauthorized cryptocurrency mining, highlight the critical need for more robust security measures [24].

To address these vulnerabilities, advancements in runtime security mechanisms are essential. Continuous trust evaluation and adaptive mitigation strategies can provide dynamic defenses, enhancing the protection of containerized microservices in ever-evolving environments. However, the research on runtime security mechanisms for MSA applications remains limited. A study revealed that only around 12% of MSA research focuses on security mechanisms, with most efforts concentrated on authentication, authorization, and encryption [27]. Repetto et al. [28] emphasize that current service mesh implementations often lack runtime trust evaluation capabilities, leaving vulnerabilities exploitable. For instance, the reliance on static access control mechanisms fails to provide the runtime adaptability needed to address emerging threats based on the changing trustworthiness of microservices [29]. Addressing these research gaps is essential to fortify MSA applications, emphasizing the need for dynamic runtime security mechanisms capable of proactively mitigating vulnerabilities and adapting to evolving threats at runtime.

Current research highlights efforts to enhance the security of MSA applications through automated vulnerability assessments, though many approaches still depend on human involvement. For example, Singh et al. [30] propose a quantitative method for security risk evaluation, while Bila et al. [31] suggest continuous vulnerability scanning with limited mitigation options. Torkura et al. [32] devise an approach for security evaluations, which requires human oversight, a critical factor in today’s cybersecurity practices [33]. Practitioners often overlook routine vulnerability checks [34] or lack the necessary expertise [35]. Integrating vulnerability assessment tools into the development workflow can be costly, as it requires skilled personnel and additional resources [35]. Tools, like Trivy [36], can scan containers for vulnerabilities but primarily operate manually and are mainly used in pre-deployment phases rather continuously during runtime [37]. This gap highlights the need for continuous scanning and monitoring of running containers in microservices at runtime. A runtime-focused approach could enable proactive vulnerability detection and mitigation, reducing reliance on manual processes and addressing vulnerabilities as they emerge.

To address these challenges in securing MSA applications, we extend our prior work, called the Service Mesh Risk-Adaptive Access Control (SMAAC) model [19] in this work. SMAAC enhances MSA security by dynamically adjusting access control policies of deployed microservices through a runtime trust evaluator (RTE) [38]. The early RTE implementation had limitations, such as a statically assigned trust metric (TM) with a default value of 100% for all microservices, which would only be decreased after unauthorized access is detected. Additionally, the initial RTE lacked automated mechanisms for dynamically updating TM values based on runtime vulnerability assessments, which identify risks posed by third-party modules and libraries. Trust management principles emphasize the need for dynamic adjustments based on behaviors and interactions [39], but current industry practices for continuous vulnerability assessments remain largely manual [40,41], reducing efficiency and leaving gaps in security. There is a need for enhancements to the SMAAC model to integrate automated, continuous trust evaluation mechanisms based on the vulnerability of deployed microservices, enabling more adaptive and resilient microservice security.

The three key contributions of SMAAC as enhanced in this work are: (1) calculating the initial TM for microservices based on vulnerability assessments to establish a baseline for managing trust, (2) dynamically adjusting the trustworthiness of microservices by increasing or decreasing their TM based on their vulnerability posture to enable holistic trust management, and (3) sharing vulnerability assessment results across the service mesh to support future self-adaptive mechanisms, such as adaptive encryption and dynamic data labeling. To evaluate these contributions, we conducted experiments using the Train Ticket MSA benchmark application [42], known for its complexity and suitability for assessing diverse research approaches. Two rounds of Train Ticket deployments—representing older and newer versions—were evaluated, with each microservice hosted as a Docker container, reflecting its widespread use in both industry and academia [43]. The containers were orchestrated using Kubernetes [17], a leading platform for scalable container management, while Istio, a widely used service mesh, securely managed microservice-to-microservice communications.

## 2. Background

Cloud-native computing, a recent advancement in cloud computing, facilitates the creation, deployment, and management of microservice architecture applications (MSAs) to meet business requirements, such as high scalability and resiliency [1]. However, handling microservice-to-microservice communications independently within each microservice can be both inefficient and complex, particularly as the adoption of MSAs continues to grow [2]. It is essential to establish controls for identification, authentication, authorization, and accounting in communications involving MSA applications, which operate in two main forms of interactions: synchronous (request-response), offering immediate replies, and asynchronous (event-driven), where responses are event-triggered [9]. It is essential to consistently apply and enforce access control policies across all interactions, including those involving services, users, and external systems [8]. To address these challenges, the cloud-native community introduced the concept of a service mesh in 2017. A service mesh consists of two primary components: the control plane and the data plane [12]. The control plane manages microservices via attached sidecars located in the data plane [12].

The current service mesh access control implementations offer a mutual authentication control to enhance the security of microservices-to-microservices communications to get encrypted and that all microservices are authenticated [44]. However, mutual authentication alone cannot identify or detect compromised microservices [19]. Attackers may exploit vulnerabilities in an authenticated microservice to launch attacks on other microservices, potentially leading to severe consequences and unauthorized activities, such as cryptocurrency mining [24], leaking sensitive encrypted data [45], and executing denial-of-service attacks against targeted organizations. Furthermore, once an attacker gains access through compromised microservices, this entry point can facilitate further escalation, allowing lateral movement across other microservices. This risk is particularly possible in environments where deployed microservices implicitly trust each other [32,46].

In the telecommunications industry, future networks, such as B5G, rely heavily on MSA and cloud-native infrastructure and technologies, such as the service mesh to create dynamic networks capable of meeting expanding business demands. In these advanced networks, core network hardware is increasingly deployed as software-defined components, eliminating traditional hardware dependencies. This shift allows for greater flexibility in the telecommunication industry to address the evolving needs of businesses and customers. Such software-defined components necessitate an infrastructure environment like cloud-native computing, which provides high scalability, resilience, fault tolerance, and robust security. The service mesh in B5G networks plays a critical role, offering essential features such as microservices discovery, observability, load balancing, security, and enhanced management capabilities [11].

However, challenges persist in these evolving B5G environments, particularly regarding the continuous monitoring of deployed microservices, which remains a significant concern [7]. Addressing such challenges is crucial to maintaining a secure B5G network posture. Researchers have highlighted the transformative potential of the service mesh for post-B5G networks, emphasizing its ability to address complex security and management needs. As Lee et al. [11] point out, the service mesh is a promising technology and will play a pivotal role in overcoming critical challenges in these next-generation networks, making it an indispensable component of future telecommunications infrastructure. However, the current implementation of the service mesh is relatively new and still maturing [47], necessitating further enhancements. One area requiring improvement is the access control capability in the service mesh. Current service mesh implementations rely heavily on role-based access control (RBAC) [29], which is often deemed inadequate for risk management because it uses static policies and does not consider the continuous finding of trustworthiness of deployed microservices. This absence of dynamic security mechanisms prevents a service mesh from adapting to evolving conditions, such as identifying and mitigating compromised microservices. Consequently, static or predefined security configurations leave deployed MSA applications vulnerable to threats, especially in advanced networks like 6G.

The zero-trust principle—“always verify, never trust”—emphasizes the need for continual evaluation of the trustworthiness of subjects, such as microservices. This approach mandates continuous validation of every access request, emphasizing strict identity verification, least privilege access, and consistent monitoring to mitigate risks from both external and internal threats. The assessment of microservice trustworthiness is critical for the effective adjustment of access control policies [48,49,50]. To mitigate these risks and potential threats effectively, a more robust mechanism for microservice-to-microservice communication is needed. One promising approach, as proposed by Sethuraman et al. [51], is the continuous monitoring of deployed microservices at runtime. By gathering insights from the application’s running environment, trust values can be dynamically adjusted [52], enabling a more adaptive and resilient access control framework.

The concept of trust evaluation was initially introduced by the U.S. Department of Defense under the term “Black Core” [53], which uses algorithms to evaluate trust by considering various factors, including access type, usage history, resource consumption, and existing policies. Currently, there is a significant research gap in runtime mechanisms for MSA applications [54], with only around 12% of existing studies addressing MSA security [27]. Most research has focused primarily on authentication, authorization, and encryption [32], leaving the area of trust management for MSAs at runtime relatively underexplored and at an early stage [48]. A recent survey by [55] found that there are few contributions related to assessing the trustworthiness of services in both service-oriented architecture (SOA) and MSA but none of these contributions were designed through leveraging the service mesh. The current service mesh architecture cannot evaluate service trustworthiness at runtime [28], indicating a pressing need for the development of trust evaluation mechanisms. As highlighted by the national institute of standards and technology (NIST), the trustworthiness of deployed microservices must be continuously assessed [56], particularly for victim microservices that have blindly granted implicit trust to compromised microservices [22,57].

**Figure 1:**
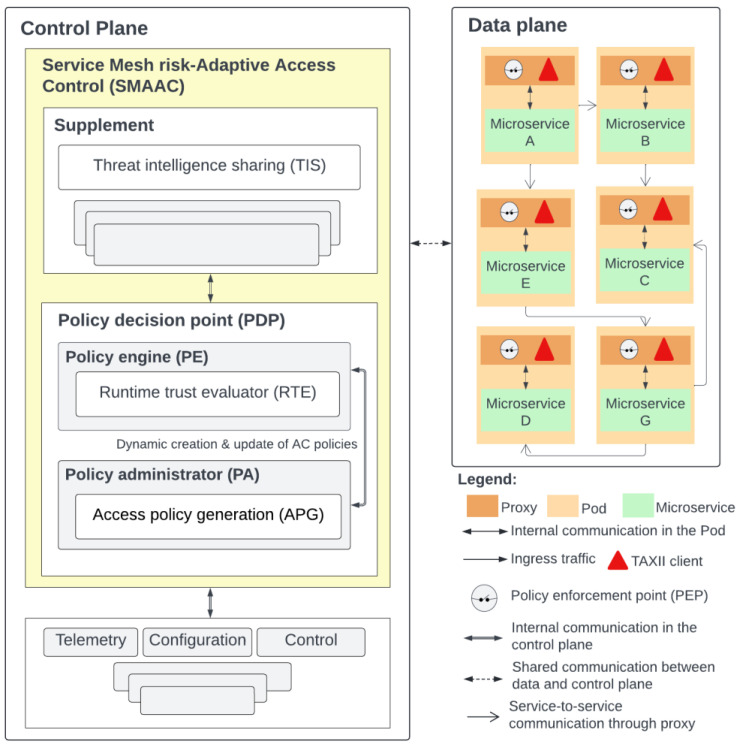
The service mesh risk-adaptive access control (SMAAC) model within a demo MSA application, which consists of six microservices [19].

To address research gaps in service mesh trustworthiness monitoring of microservices at runtime, we proposed an initial approach called Service Mesh Risk-Adaptive Access Control (SMAAC) [19] as shown in Figure 1. SMAAC presents a dynamic access control policy model that adjusts microservice interaction policies based on deployed microservices’ trustworthiness at runtime. These policies are dynamically changed in response to detected malicious behavior, especially when access attempts to unrelated microservices occur. This strategy aims to reduce attack surfaces and limit harmful interactions, thereby allowing only necessary and trusted communications. SMAAC includes a policy decision point (PDP) with a runtime trust evaluator (RTE) [38], assigning an initial trust metric (TM) of 100% to microservices, which decreases upon identification of malicious behavior coming from a microservice through a pricing mechanism. Moreover, SMAAC features an initial threat intelligence sharing (TIS) [58] capability for threat data exchange within the service mesh, existing as an additional component. SMAAC provides an access policy generation (APG) [19] capability to dynamically formulate access control policies based on each microservice’s TM, promoting both least privilege and adaptive policies. APG has been further refined to automate compliance validation via a compliance-as-code language [59], ensuring adherence to requirements defined by the owners of microservices.

SMAAC possesses inherent advantages, yet its early consideration faced constraints in delivering holistic trust management. To enhance efficacy, it is imperative to establish an initial TM based on a vulnerability assessment instead of assigning a default TM value of 100%. Furthermore, trust should be dynamically modified—both increased and decreased—based on insights from the operational context of the MSA application [39]. Additionally, SMAAC must improve TM values for microservices demonstrating positive actions, such as addressing vulnerabilities in modules and libraries within the deployed MSA application. The trustworthiness evaluation of microservices must encompass criteria like the scanning of each container image used by each microservice [60], which can be assimilated into vulnerability assessment processes to facilitate a more precise and adaptive trust management framework within the service mesh.

Overall, conducting a vulnerability security assessment is essential for deployed MSA applications, particularly given that research indicates an average of 180 vulnerabilities in MSA applications compared to just 39 in monolithic applications [25]. This increase in vulnerabilities is primarily attributed to the potential use of unsecured container images for microservices [61]. Deployed MSA applications, and containers are considered the default method [35,62]. Compromised containers can be targeted through supply chain attacks, which represent a significant threat, as noted in a recent publication by the National Institute of Standards and Technology (NIST) [63]. Homogeneous MSA applications, characterized by a high degree of code reuse, may exacerbate security risks [64]. Therefore, conducting vulnerability assessments is crucial not only for securing the containers of microservices but also for ensuring compliance with standard security guidelines [65]. However, one significant issue is that current industry practices for vulnerability assessment, particularly performed manually are often ineffective, especially for large numbers of containers [40,41]. Vulnerability assessment processes include searching for security issues in the deployed environment and determining the severity of the issues, which then help to build controls to safeguard the deployed application environment [66].

**Figure 2:**
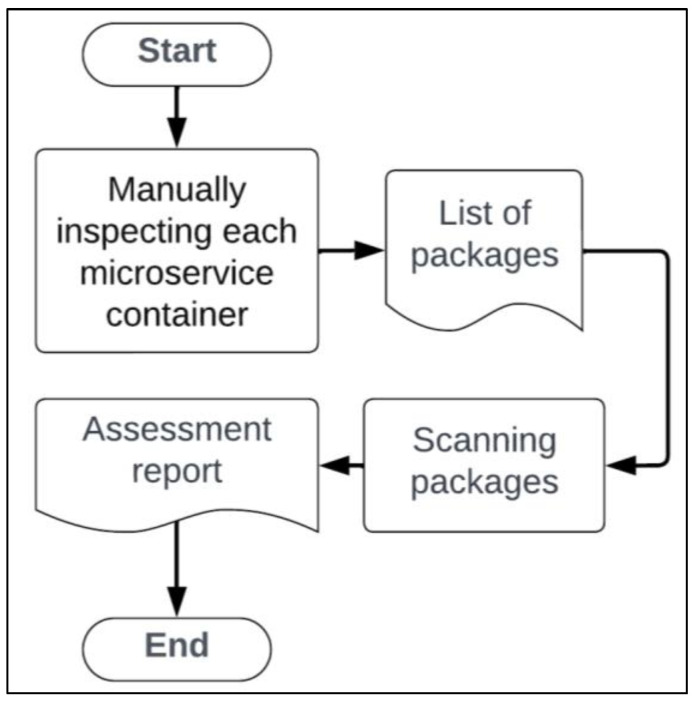
The container vulnerability scanning tasks followed as current practices [67].

As demonstrated by Javed et al. [67], a manual container vulnerability assessment consists of three main steps, as shown in Figure 2. The first step involves the scanning tool examining the packages within a container image by analyzing their version numbers and names. In the next step, the information collected from the previous process is cross-referenced with a vulnerability database. Once a match is found, a vulnerability assessment report is generated, which requires human interpretation. Human oversight presents a significant challenge [33], as personnel often overlook regular vulnerability checks or may lack the expertise needed for continuous assessments [34].

Integrating effective vulnerability assessment processes into an organization’s environment can be costly, primarily due to the expenses involved in hiring skilled personnel to operate and interpret the vulnerability assessment tools’ results [35]. These factors can hinder the effectiveness of vulnerability management efforts, leaving organizations exposed to security threats. Attackers can exploit well-known vulnerabilities, such as those documented in the common vulnerabilities and exposures (CVE) database, in deployed systems like containers [68] where attackers have identified vulnerabilities in over 50,000 containers, which they have subsequently exploited for cryptocurrency mining [24]. Delayed vulnerability checks and patching could lead to the use of insecure containers, which can expose hosted services to unauthorized access and increase the risk of privilege escalation attacks.

As MSA applications increasingly rely on containers, the risk of compromised containers is likely to increase with their growing adoption. Typically, MSA applications are deployed in orchestrated environments that utilize solutions such as Kubernetes [17]. Kubernetes operators can leverage vulnerability scanning to inspect deployment containers before they are incorporated into a cluster. However, this process often occurs manually [37]. Kaiser et al. [69] highlight several container vulnerability scanning tools, including Snyk [70], Trivy [36], Clair [71], and Anchore [72]. Among these, Trivy is particularly recognized for its effectiveness [37], demonstrating high coverage for image issues [73] and consistently detecting vulnerabilities [74]. However, the manual execution of these tools during runtime remains relatively immature, facing challenges in timely detection and vulnerability patching.

Ongoing scanning is essential for maintaining security, rather than relying solely on a one-time assessment before deployment. Automated solutions can address critical gaps in vulnerability management, reducing risks associated with compromised containers. Continuous and automated vulnerability scanning of running containers is urgently needed [75] to enhance visibility and management [76] for deployed MSA applications, especially for 6G networks. Automated vulnerability assessment and remediation can help detect vulnerabilities early in microservices at runtime and determine appropriate mitigation strategies, particularly in production environments, to prevent potential attacks [31].

Docker, introduced in 2013, simplifies the conceptualization, execution, and management of applications [77]. While Docker simplifies environment sharing, it lacks sufficient protection against known vulnerabilities in images, potentially compromising MSA application environments. Compared to virtual machines (VMs), containers share a single operating system, improving performance over VMs but introducing unique security challenges [77]. According to Open Worldwide Application Security Project (OWASP) [78], a primary threat to MSA applications is the potential for attackers to exploit container privileges to target other microservices. Studies indicate that 92% of container images contain unpatched vulnerabilities [12], making them attractive targets for exploits like cryptocurrency mining [24]. In a study conducted by Winkel et al. [79], a security architect who works at Docker highlights the importance of conducting a risk analysis of containers, noting that they are vulnerable to malicious activities and cannot always be trusted. Containers continue to pose security issues, with a significant proportion (one-fifth) of container images containing vulnerabilities due to neglect in performing updates and available patching [80].

To improve vulnerability assessment methods for deployed containers, existing research highlights various approaches. Joshi et al. [30] suggest using Common Vulnerability Scoring System (CVSS) metrics for quantitative risk evaluation [81]. CVSS allows organizations to prioritize their vulnerability management efforts based on risk. The CVSS scoring system includes several metrics, each assigned a specific value. For example, the attack vector metric within the CVSS scoring system, which measures the ease of remote exploitation of a vulnerability, is assigned a score of 0.85. These individual metrics scores are aggregated to produce a cumulative value representing the overall CVSS score for each vulnerability found. While CVSS provides a standardized framework for assessing vulnerabilities, its approach focuses on individual vulnerabilities rather than aggregating risk scores across all vulnerabilities that could exist in each deployed microservice.

Another study by Bila et al. [31] proposes continuous scanning and mitigation strategies, such as deleting or quarantining vulnerable pods by checking if a deployed container continues a vulnerable package. The limitation of their work could lead to the loss of valuable data or result in complete access denial, even in cases where the vulnerabilities are not considered high risk. Also, it is not based on the service mesh architecture. In 2017, ref. [32] introduced a methodology for ongoing security evaluation in MSA applications, utilizing dynamic document stores for vulnerability identification. Their expanded work in [82] proposed the Cloud Aware Vulnerability Assessment System (CAVAS) to dynamically execute vulnerability assessments but did not leverage the service mesh for efficiency. Further advancements [64] proposed a solution using the Anchore [72] as a vulnerability scanning tool and introduced a security risk (SR) metric based on CVSS scores, which sums vulnerability scores divided by the total number of vulnerabilities. They also applied a shrinkage estimator for microservice dependencies. However, the study lacks a comprehensive evaluation of the SR and does not assign severity weights to CVSS scores or categorize severity effectively. Another study presented by [25] relies on Clair [71] to propose a framework that aids in discovering and analyzing security flaws in container images. However, the results of the analysis require human intervention for interpretation. Similarly, Ibrahim et al. [68] introduced automated attack graph mechanisms, but these also require human intervention for data interpretation.

The study in [45] addresses a notable gap in automated security detection mechanisms within cloud-native environments. This gap prompted the development of KUBEHOUND [45], a tool designed to identify potential security issues in cloud-native services. While KUBEHOUND contributes to security by analyzing deployed services, it has some limitations. It requires access to the source code of each microservice, which may not always be feasible or practical in production environments. Additionally, KUBEHOUND cannot scan for known vulnerabilities, leaving a gap in comprehensive vulnerability management. Majumder et al. [83] proposed a methodology to improve container security by performing vulnerability scans on container images. Their approach leverages vulnerability scanning tools such, as Clair [71] and Trivy [36], to generate a vulnerability score, called v-score, that reflects the security risk associated with a given container image. The v-score is then compared to a defined threshold risk score or t-score to determine if the container image should be allowed or blocked from deployment. If the v-score is lower than the t-score, the image upload is blocked, ensuring only those that meet a specific security standard proceed. However, there are notable limitations in the methodology. The study lacks transparency in how the proposed scores are calculated, raising questions about the scoring criteria and their reliability. The approach is limited to pre-deployment stages, meaning it does not account for vulnerabilities that might emerge at runtime, a critical gap in continuous security monitoring.

The studies in [84,85] provide important advancements in container and microservices security, revealing certain gaps regarding the integration of service mesh technology. Abdulsatar et al. [84] introduced the CyberWise Predictor, a framework designed to detect and assess vulnerabilities in deployed containers for MSA applications. This framework follows a four-phase process that includes scanning applications with Trivy [36] to identify vulnerabilities, matching those vulnerabilities with the national vulnerability database (NVD) [86] to gather relevant metrics, perform data cleaning using deep learning to address any missing information, and conduct a risk assessment to evaluate the overall security risk. Kermabon et al. [85] proposed Phoenix to protect containers from zero-day vulnerabilities by analyzing system calls. Despite its focus on zero-day threats, Phoenix does not incorporate service mesh technology into its security framework. Both approaches miss the opportunity to leverage a service mesh layer, which holds significant potential for enhancing security especially as MSA gains traction. As noted by Berardi et al. [87], while there are numerous studies focused on securing microservices and references to blockchain technologies, there remains a notable lack of attention given to the service mesh. This oversight is particularly concerning given the anticipated rise in service mesh adoption alongside the growth of MSA.

Although service mesh technology is still evolving [88], it holds significant potential for security advancements, particularly when enhanced with automated vulnerability assessments. Automating vulnerability assessments within the service mesh substantially reduce the risk of compromised microservices, as continuous monitoring helps detect vulnerabilities as they arise, minimizing exposure time. Recent research underscores that deploying a service mesh not only secures microservice-to-microservice communications but also strengthens authentication and authorization measures for each interaction [89]. This enhanced security is essential in distributed applications, where every microservice interaction could represent a potential entry point for attackers. Moreover, the service mesh can play a crucial role in trust evaluation and dynamic access control, allowing for adaptive security policies that respond to changing conditions due to many operational reasons. One reason is the service mesh’s uniquely capability to intercept all microservice-to-microservice traffic [14] provides deep visibility into communication patterns and data flows within deployed MSA applications, forming a solid foundation for detecting unusual behaviors or suspicious activities.

Furthermore, by leveraging sidecars in a deployed service mesh that functions as policy enforcement point (PEP), a service mesh provides granular control over the policies governing each microservice interaction [15]. Thus, policies can be enforced at every endpoint, dynamic encryption methods can be applied and so on to ensure strict compliance with security standards and reduce the risk of unauthorized access or data leaks. Also, the service mesh enables observability through metrics, logs, and traces to show insights needed to identify trends and detect threats early. Together, these features make a service mesh a powerful addition to microservice security frameworks, effectively addressing the inherent complexity and security challenges of deployed MSA applications.

In the current industry practice, automated continuous vulnerability scanning is typically enforced through an admission controller within the deployed orchestration solutions [17] that can be configured to scan new deployments before they go live in production. While this approach is effective for pre-deployment security, such as when a new application deployment is added, it does not address runtime operations of deployed application and requires pre-built controllers. Additional best practices include container image integrity checks to ensure that only trusted images are deployed, software bill of materials (SBOM) analysis for documenting all components in a software product to assess vulnerabilities, and continuous monitoring of CVEs along with vulnerability scanning to regularly check for known vulnerabilities in active systems [78]. An SBOM assists in identifying vulnerabilities during incident response, ensuring compliance and licensing checks, and tracking dependencies effectively. Software composition analysis (SCA) tools are used to evaluate third-party dependencies and are integrated into development and deployment pipelines for vulnerability detection [73]. However, these tools often lack comprehensive risk assessment capabilities and do not operate in real-time during runtime.

The absence of vulnerability detection and patching of microservices during runtime can expand attack surfaces, making systems more vulnerable to exploits that target either the container kernel or its internal modules. Research indicates there is a significant delay in identifying and addressing vulnerabilities, with a mean time to identify (MTTI) of seven days and a mean time to remediate (MTTR) of up to 26 days in some cases [68]. This delay allows attackers to exploit known vulnerabilities—typically those listed in the CVE database—within deployed microservices before patches are applied. It is clearly stated that the current security tools often fail to fully identify asset-related security issues, as noted by [35]. Therefore, effective security management and the prioritization of vulnerabilities are crucial [73]. The Center for Internet Security (CIS) advocates for automated assessments to detect and address vulnerabilities, particularly for deployed microservice containers [90]. The goal of implementing automated vulnerability assessments is to detect issues and initiate self-protection measures to reduce the attack surface exposed by malicious services.

## 3. Approach

This study presents a trust evaluation mechanism for deployed MSA applications, leveraging automated vulnerability risk assessments. It assigns an initial and continuous trust metric (TM) to each microservice based on the vulnerability levels of its third-party modules and libraries. Our approach operates within MSA applications without human intervention, addressing limitations in related work. It automates vulnerability scans of microservice images and dynamically adjusts a TM based on their security posture, enabling real-time trust evaluation with minimal input. Security analysts must define the risk tolerance of the MSA application’s owning organization and severity weights for vulnerability categories. Once deployed with the service mesh, it requires no additional configuration beyond the standard management of cloud-native technologies, such as the service mesh and orchestration solutions.

In this work, the TM for each microservice is derived from vulnerabilities identified in the microservice’s third-party modules and libraries, utilizing Trivy (version 0.57.0) [36]—a widely used open-source vulnerability scanning tool recognized for its extensive CVE database and accessibility [91]. Trivy enables the identification and classification of vulnerabilities based on severity levels and provides vulnerability metrics using the Common Vulnerability Scoring System (CVSS), a standard widely adopted by the security community for assessing software vulnerability posture. However, Trivy, like other similar vulnerability scanning tools, lacks automation capabilities for runtime vulnerability assessment of MSA applications within a service mesh environment.

Unlike prior works, such as [84,85], which do not fully address emerging technologies like the service mesh, our proposed mechanism integrates directly with the service mesh to calculate the TM for each microservice based on varying CVSS scores and their impact levels. Our mechanism also bridges a gap in existing research by performing runtime vulnerability scanning, addressing limitations where vulnerability assessments are limited to the development and deployment phases [37,68]. Moreover, this study provides a detailed methodology for calculating the TM from identified vulnerabilities, addressing gaps noted in [83], where the scoring process and calculations were insufficiently explained. Our approach tackles automation challenges prevalent in existing works, such as manual image uploads for scanning [77] and manual review of vulnerability results [92]. By automating the retrieval of deployed microservice images and the processing of vulnerability reports, our approach streamlines the calculation of the TM for each microservice, enhancing scalability and efficiency in MSA security management.

Our approach integrates enhances the SMAAC model (see Section 2) to address its limitations through five key processes: (1) discovering microservices and their container images at runtime, (2) scanning container images for vulnerabilities, (3) assessing vulnerabilities for each microservice, (4) evaluating microservice trustworthiness, and (5) sharing vulnerability assessments to enable future dynamic mitigation strategies, such as adaptive encryption. The approach operates continuously, adhering to the zero-trust principle “always verify, never trust”, which is critical for dynamic cloud-native environments, especially in 6G networks [93]. The first two processes form the foundation for automated security assessments, ensuring precise identification of all active microservices within the TIS of SMAAC (see Section 2). The third and fourth processes, within the RTE of SMAAC (see Section 2), establish and maintain the TM based on vulnerability assessments. Process five enhances collaborative security by enabling threat and vulnerability data sharing, which is integrated into the TIS of SMAAC.

**Figure 3:**
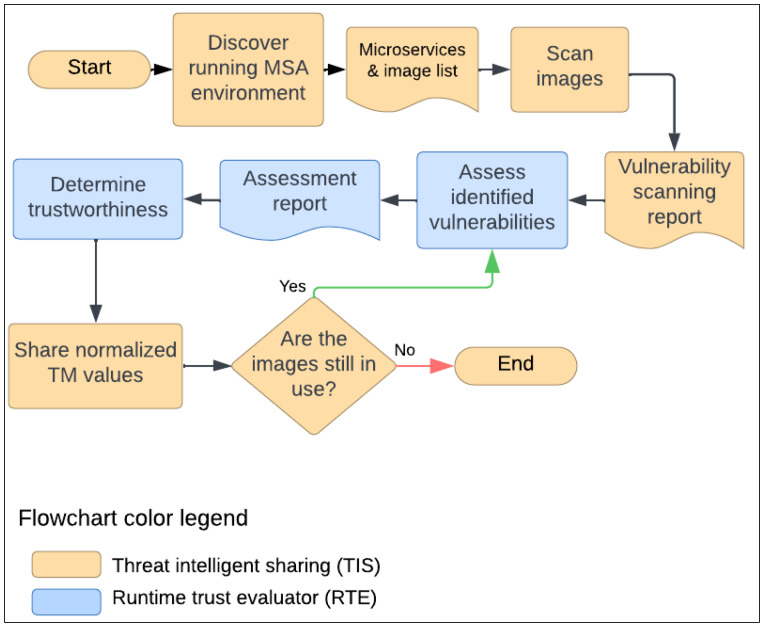
The proposed approach has five processes colored in two colors: orange and blue. The orange color is for the TIS [58], including the start and end, three processes, and condition. The two blue processes belong to the RTE [38].

As illustrated in Figure 3, the first process, titled “Discover running MSA environment”, is initiated by the TIS in the start icon in orange. The first process focuses on identifying active microservices using an orchestration solution, such as Kubernetes [17], which provides built-in microservice discovery capabilities. This process is initiated by the TIS using Kubernetes’ Python software development kit (version 31.0.0) [17], enabling the detection of deployed and running microservices. In addition to identifying all deployed microservices, this process generates a comprehensive list of each microservice’s containers, including their image names and repository URLs. The result is a complete, machine-readable record, referred to as the “Microservices & Image List”, which details all microservices along with their associated image names and URLs.

The second process, titled “Scan images”, involves scanning the list of images and packages identified in the previous step. During this process, each microservice’s modules and libraries are scanned for vulnerabilities using the Trivy vulnerability scanning tool [36]. Trivy categorizes vulnerabilities into critical, high, medium, low, and unknown levels. For this work, we focus on vulnerabilities classified as critical, high, medium, and low severity, as unknown vulnerabilities cannot be evaluated. The outcome of this process is a machine-readable vulnerability assessment report, detailing each microservice’s vulnerable modules and libraries, along with their associated severity levels, represented by CVSS scores.

The third process, titled “Assess identified vulnerabilities”, involves a detailed analysis of each microservice’s vulnerability assessment report to derive an accurate TM based on runtime vulnerability risk. This derivation is achieved by leveraging the list of vulnerabilities identified in the previous process. This step is integrated into the RTE, which we extended in this work to continuously calculate the vulnerability value (*Vul_s_*) for each deployed microservice, *s*, based on the collected vulnerability assessment reports. As shown in Equation (1), the *Vul_s_* is calculated by summing the CVSS scores of the vulnerabilities found in each microservice’s libraries and modules given severity categories. Each CVSS score is then multiplied by a weight (*W*) assigned to the respective CVSS severity category—low, medium, high, or critical. The *W* for each severity category is predetermined by the risk team of the MSA application’s owning organization to align with the organization’s risk tolerance, ensuring the weights accurately reflect the organization’s risk management priorities.

(1) $$\;{Vul}_{s}=[\sum_{category}{(CVSS}_{category}\times W_{category})](s)$$

Here *CVSS_category_* represents the sum of CVSS scores within each severity category: low, medium, high, and critical. Each total is then multiplied by the corresponding weight, (*W_category_*), for the same category. In this work, the weights are defined as follows: the weight for low CVVS (*W_low_*) is set at 0.10, the weight for medium CVVS (*W_medium_*) is set at 0.20, the weight for high CVVS (*W_high_*) is set at 0.30, and the weight for critical CVSS (*W_critical_*) is set at 0.40. The weights can be determined by the security analysts or obtained from an open dataset, if available. However, this could increase performance overhead due to the varying processing requirements of the dataset. Therefore, it is advisable to manage such configurations independently or accept the potential performance impact if the business requires dataset integration. This research relies on the use of a configuration file with initial default settings that reflects the risk tolerance of the owner of the deployed MSA application.

The next process, titled “Determine trustworthiness”, calculates the TM for each microservice using the scaled equation proposed in Equation (2), which incorporates the computed *Vul* for all microservices. This process operates in a continuous loop to ensure that the TM is dynamically recalculated at runtime. To normalize the TM across microservices, the calculated values are scaled to fall within a range of 0 to 100, providing a clear and consistent measure of each microservice’s trustworthiness.

(2) $${TM}_{s}=100-\frac{\left({Vul}_{s}-MinVul\right)}{\left(MaxVul-MinVul\right)}\times 100$$

For each microservice s, *MinVul* represents the minimum *Vul_s_* identified by our proposed mechanism at runtime among all deployed microservices, while *MaxVul* represents the maximum *Vul_s_* identified among the same set of microservices. These values are used to scale the calculated TM for each microservice within the range of 0 to 100, ensuring consistency and comparability across different microservices. To demonstrate Equations (1) and (2), an example is provided in Table 1 using a DEMO MSA application consisting of four microservices. This illustration showcases the calculation and scaling process of TM in a practical context.

The next process, titled “Share normalized TM values”, facilitates the distribution of the TM values across all microservices within the service mesh components. This sharing enables the implementation of future self-adaptive remediation strategies, such as adaptive encryption, dynamic service and data labeling, traffic routing, and other potential solutions. The process is executed through by enhancing TIS within the SMAAC model to share both initial and continuously updated TM values along with vulnerability assessment reports in a machine-readable format. The result of this process is to update the TIS repository, which already has the capability to leverage two well-known frameworks for threat sharing: structured threat information expression (STIX) [94] and trusted automated exchange of intelligence information (TAXII) [95]. However, in this work, we focused on regularly updating the *TM_s_* and *Vul_s_* for each microservice *s*.

The next condition of the proposed approach, titled “Are the Images Still in Use?”, operates within the TIS in a continuous loop to ensure that each active microservice container image is regularly scanned for vulnerabilities. This ongoing process is essential for maintaining the security posture of deployed microservices by promptly identifying and mitigating potential risks. Additionally, this condition dynamically adjusts TM values to reflect positive behavior, such as the successful patching of a vulnerable third-party module—a key focus of this study. By rewarding such actions, the system fosters continuous security improvements. If the condition is met, the TIS triggers the RTE to re-calculate the TM automatically, addressing the RTE’s previous inability to perform continuous TM updates, as highlighted in the background section. Otherwise, the process is terminated by the TIS, as indicated by the orange end icon, because the image is no longer in use. However, when a new image is added to a microservice, the same process is repeated automatically, and the TIS initiates such requests to perform dynamic trust evaluation, as represented by the orange start icon.

## 4. Evaluation

To evaluate our proposed approach, we utilize the Train Ticket MSA [42] application, a widely recognized MSA benchmark in cloud-native security studies due to its complexity. The evaluation environment incorporates Kubernetes [17] for orchestration and Istio [44] as the service mesh platform, ensuring the adoption of widely used cloud-native technologies. In addition, this setup simulates real-world MSA application deployment scenarios, aligning with best practices in both the research community and academia. The Train Ticket MSA consists of over 40 microservices that facilitate essential operations within train ticketing and management systems. The evaluation environment incorporates Kubernetes [17] for orchestration and Istio [44] as the service mesh platform, ensuring the adoption of widely used cloud-native technologies. This setup simulates real-world MSA application deployment scenarios and aligns with best practices recognized in both the research community and academia.

**Figure 4:**
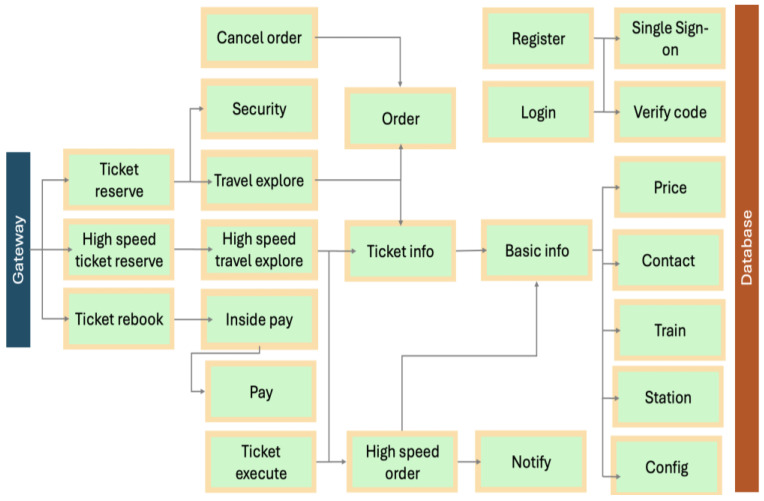
A basic and practical architecture of the Train Ticket [42] adopted from [96].

Adapted from [96], a basic and practical architecture of the Train Ticket MSA as illustrated in Figure 4, delivers a wide range of functionalities. As shown in the figure, the Train Ticket MSA provides an excellent user experience for both customers and station personnel through a gateway (one the left) and a database for storing changes and updates (on the right). Customers can seamlessly sign in to search for available trains, reserve tickets, modify bookings, process payments, and manage their travel plans. For station personnel, the Train Ticket MSA monitors train schedules, handles payments, notifies customers, and manages bookings.

We conducted a two-phase evaluation of our approach using different versions of the Train Ticket MSA application. Phase one used an older version of Train Ticket (0.0.4) as shown in Table 2 to establish a baseline, while phase two tested a newer version of Train Ticket (1.0.0) as shown in Table 3 to demonstrate adaptation to changing MSA applications environments. Both phases utilized five microservices with distinct base images to ensure robustness. This evaluation highlights the approach’s effectiveness in identifying vulnerabilities, calculating *TM_s_*, and enabling dynamic trust evaluation through seamless data sharing across the service mesh. In this evaluation, the proposed approach automates risk assessment for deployed microservices by evaluating their trustworthiness through runtime vulnerability assessment. The process begins by identifying five selected microservices and their associated container images. The five selected microservices are: *admin-order*, *avatar*, *news*, *ticket-office*, and *voucher*. Once the identification happens, each container image is then scanned for vulnerabilities. For example, the *admin-order* microservice was found to have 65 low, 174 medium, 163 high, and 76 critical vulnerabilities. A full analysis was conducted for the other microservices, as detailed in Table 2. Next, the proposed approach calculates *Vul_s_* and *TM_s_* for each microservice *s* using CVSS scores and the proposed two equations. Using Equation (1), the *Vul_s_* is determined by multiplying each CVSS score by its designated weight. For instance, the *Vul_admin-order_* for the *admin-order* microservice is calculated as (65×0.1)+(174×0.20)+(163×0.30)+(76×0.4)=120.60 as detailed in the approach section.

Following this, the proposed approach applies Equation (2), as detailed in the approach section, to calculate the *TM* for each microservice using their respective *Vul_s_.* This process involves determining the minimum and maximum *Vul* values refer as *MinVul* and *MaxVul* across all deployed microservices. For example, continuing with the *admin-order* microservice in the older version, the *MinVul* is 27.70, and the *MaxVul* is 1649.6. Using these parameters, the *TM_admin-order_* is calculated as follows: (100−((120.6−27.7) / (1649.6−27.7))×100 which gives the value of 94.27. The same process is repeated for the other microservices, with results summarized in the Table 2.

To demonstrate the effectiveness of our approach in a dynamic environment where microservices are frequently updated, our proposed mechanism operates in a continuous loop, automatically checking each microservice and their container images. When a patch is applied, the process is re-executed to update *Vul* and *TM* values for all microservices without human intervention, as shown in Table 3. In the process, the calculation of the *MinVul* and *MaxVul* is repeated. In the updated version of the Train Ticket MSA, the *news* microservice still holds the *MinVul*, but the *MaxVul* shifts from the *voucher* microservice to the *avatar* microservice in this new version of the Train Ticket. In this evaluation, the TM for all microservices is then recalculated. Among these, the *TM_voucher_* for the *voucher* microservice increases by 6.17 due to the reduction in vulnerabilities within the microservice. Also, the *TM_ticket-office_* decreases significantly from 57.00 to 7.11. The *TM* for the other microservices, except the *news* microservice which remains unchanged, is adjusted accordingly.

**Figure 5:**
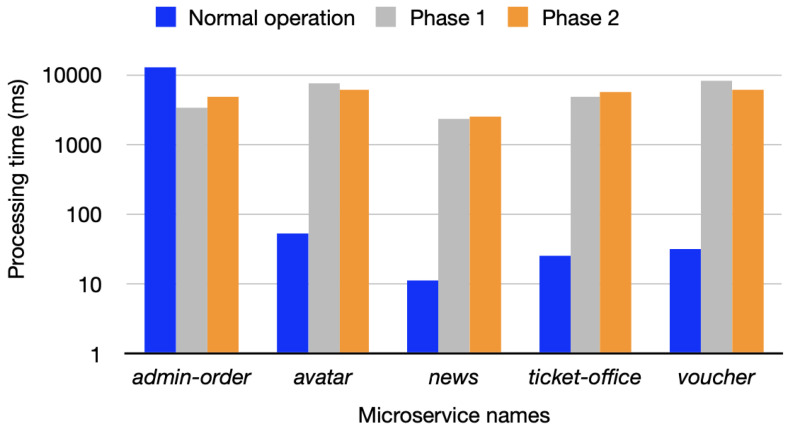
Performance evaluation of processing times for five microservices during normal operation (blue), Phase 1 (light gray), and Phase 2 (orange).

As shown in Figure 5, we evaluated our approach by measuring runtime processing times for the evaluation of Phase 2 of the Train Ticket system without imposing any security mechanisms (blue) and with our proposed mechanisms in Phase 2 (orange). We included a performance evaluation for Phase 1 of the old Train Ticket system as the third bar in light gray, but did not evaluate the Phase 1 original system. We conducted the performance testing using a performance tool (version 1.0.0) [97] based on the modern load testing framework called Locust (version 2.32.1) [98], simulating 100 users (peak concurrency) with 10 users spawned per second. Processing times were measured for each microservice, with the *admin-order* microservice exceeding 10,000 ms under normal operation, while others stayed below 100 ms. Under our proposed security mechanisms during Phase 2, results showed the *voucher* microservice had the highest processing time and the *news* microservice the lowest. This trend persisted in Phase 1, though *admin-order*, *news*, and *ticket-office* processing times slightly decreased, while *avatar* and *voucher* increased. While these findings highlight the need for further optimization of our proposed security mechanisms, the evaluation identifies that there is a similar performance associated with each microservice when the security mechanism is put in place. This evaluation will inform future work to identify how to align the security mechanism more closely with original service performance.

## 5. Discussion and Result

The proposed approach empowers the service mesh to dynamically monitor and evaluate the trustworthiness of microservices through a runtime vulnerability assessment. The proposed mechanism continuously identifies and analyzes potential weaknesses in deployed microservices, significantly enhancing the overall security of deployed MSA applications. Detecting vulnerabilities and adjusting trustworthiness scores, accordingly, helps maintain a robust security posture throughout the lifecycle of the microservices. The proposed mechanism continuously monitors and assesses microservices’ security is especially crucial for industries demanding high-security standards, such as B5G, and next-generation environments such as 6G networks.

At its core, our proposed mechanism dynamically assigns a TM to each microservice, continuously based on the results of runtime vulnerability assessments. When risks or vulnerabilities are detected, the TM is lowered, signaling the need for prompt intervention. This runtime and automated evaluation provide organizations with more insights into the security posture of their microservices, enabling timely and informed decision-making where it paves the way for future proactive self-adaptive controls, ensuring secure operations even in the most demanding environments.

In our evaluation, we demonstrate the effectiveness of the proposed approach in dynamically adjusting the TM of deployed microservices within the Train Ticket MSA application. As microservice container images are updated, vulnerabilities may increase or decrease based on the nature of third-party modules and libraries embedded within them. Consequently, the TM dynamically reflects these changes, increasing or decreasing at runtime according to the evolving security posture of the microservice. This ensures that the TM remains aligned with the actual security status of the microservices in production. 

Compared to the five related works shown in Table 4, our approach also performs vulnerability scanning by leveraging a vulnerability scanning tool namely Trivy. However, our proposed approach stands out by uniquely adhering to the zero-trust principle of ‘always verify, never trust,’ enabling the dynamic trust evaluation of deployed microservice container images. Our approach facilitates trust evaluation based on the vulnerability levels of deployed microservices, which is seamlessly integrated into the service mesh.

The advantages of our proposed approach address critical gaps in microservices security research by introducing an efficient runtime trust evaluation, paving the way for advanced self-adaptive measures such as dynamic encryption. This enhances data confidentiality and integrity when the trustworthiness of a microservice declines. Furthermore, our approach could facilitate dynamic data classification based on vulnerability assessments, which is crucial for complex and evolving systems that require high adaptability and resiliency.

The approach is not without limitations. One disadvantage of our approach is the performance overhead. As shown in the performance evaluation in Figure 5, it takes less than 10,000 ms to complete the trust evaluation, which could introduce delays in microservice-to-microservice communication, particularly in industries requiring real-time operations with no tolerance for delays, such as emergency services. Future work will focus on optimizing performance in such environments and incorporating additional features to address these challenges. Another disadvantage of the proposed approach is its design complexity. However, once integrated into the MSA application environment and deployed as part of the service mesh, it will no longer require human involvement. It is specifically designed to address this challenge, unlike related works that rely heavily on human intervention to manually upload microservice container images, review vulnerability reports, and decide whether to accept or reject malicious images.

Future work will investigate enhanced automation by enabling runtime correction of malicious libraries in MSA applications, including updating and deploying secure versions of deployed microservices libraries and modules. A machine learning model will be integrated to predict unknown vulnerabilities that show previous high-risk libraries, preventing their use in production. Additionally, we will investigate integrating our approach with cybersecurity tools like risk management, to determine how incident management systems can streamline handling vulnerable libraries and modules and provide real-time warnings to organizations using the same libraries, fostering proactive security collaboration.

## 6. Conclusions

Cloud-native computing with the service mesh as a dedicated infrastructure layer enhances microservice architecture applications (MSAs), providing significant advantages like secure communication, high scalability, and efficiency. These advantages are crucial in Beyond 5G (B5G) environments such as Sixth-Generation (6G) networks. Nonetheless, this shift brings notable security issues, particularly concerning microservices utilizing third-party components. Securing MSA applications necessitates adherence to zero-trust principles, emphasizing continuous verification of third-party components used in deployed microservices. This research addresses a gap in existing service mesh implementations by proposing a runtime trust evaluation mechanism based on a continuous vulnerability assessment to ensure the reliability of microservice operations. Validation of the approach was conducted using the Train Ticket MSA application, the most complex MSA benchmark involving technologies Docker, Kubernetes, and Istio. Experimental findings indicated improvements in microservice trustworthiness findings, illustrating the enhanced service mesh framework’s potential in advanced network settings.

Future work will address some of the current limitations, including evaluating the proposed approach in real- or near-real-world complex 6G networks. Additionally, the proposed approach will be extended to detect unknown threats. One mechanism that will be studied is the use of a machine learning model to identify new attack scenarios. Another area for future work is integrating the proposed solution with other security systems to reduce the need for security analysts to set initial configuration parameters, such as the weight of vulnerability categories. This could be achieved by automating the process more effectively, with solutions like a risk management system from the MSA-owning organization. One possible future work is using the generated microservice trust metric (TM) to allow user input to choose the most secure services, such as selecting from multiple 6G network providers.

## Figures and Tables

**Table 1:** An Illustration of applying *Vul_s_* and *TM_s_* equations for a DEMO MSA application consists of four microservices named as A, B, C and D.

Microservice	Total CVSS Scores Based on Their Categories				*Vul_s_*	The Initial Value of *TM_s_*
	Low	Medium	High	Critical		
A	220	50	100	30	74 (*MinVul*)	100.00
B	1300	600	100	50	300	61.21
C	900	1500	222	500	656.6 (*MaxVul*)	0.0
D	109	99	15	400	195.2	79.20

**Table 2:** (Phase 1) A detailed list of five selected microservices, along with the total count of CVEs in each category (low, medium, high, and critical), is provided. Additionally, the proposed approach continuously calculates the initial *Vul_s_* and *TM_s_* for each microservice in this older version of the Train Ticket (version 0.0.4).

Microservice	Total CVSS Scores Based on Their Categories				*Vul_s_*	The Initial Value of *TM_s_*
	Low	Medium	High	Critical		
*admin-order*	65	174	163	76	120.60	94.27
*avatar*	1591	3701	1604	167	1447.30	12.47
*news*	1	31	42	22	27.70 (*MinVul*)	100.00
*ticket-office*	511	1354	1055	218	725.6	57.00
*voucher*	1599	4092	1971	200	1649.6 (*MaxVul*)	0.0

**Table 3:** (Phase 2) A detailed list of five selected microservices, along with the total count of CVEs in each category (low, medium, high, and critical), is provided. Additionally, the proposed approach continuously calculates the continuous *Vul_s_* and *TM_s_* for each microservice in this newer version of the Train Ticket (version 1.0.0).

Microservice	Total CVSS Scores Based on Their Categories				*Vul_s_*	The Continuous Value of *TM_s_*
	Low	Medium	High	Critical		
*admin-order*	65	174	163	76	120.60	91.66
*avatar*	969	3086	1270	117	1141.90 (*MaxVul*)	0.00
*news*	1	31	42	22	27.70 (*MinVul*)	100.00
*ticket-office*	906	2830	1203	113	1062.70	7.11
*voucher*	931	2842	1219	115	1073.20	6.17

**Table 4:** Comparison of our approach with related works in terms of vulnerability scanning, zero-trust principles, trust evaluation, and service mesh integration. An empty circle denotes that the approach does not support the feature, a half-filled circle indicates partial support, and a full circle signifies full support.

Approach	Vulnerability Scanning Applied	Trust Evaluation Mechanism Applied	Zero-Trust Applied	Service Mesh Based
*Kwon, S. and Lee, J.-H* [77]	⬤	◯	◯	◯
*Brady et al.* [92]	⬤	◯	◯	◯
*Javed, O. and Toor, S* [67]	⬤	◯	◯	◯
*Majumder et al.* [83]	⬤	◑	◯	◯
*Abdulsatar et al.* [84]	⬤	◯	◯	◯
*Torkura et al.* [32]	⬤	◑	◯	◯
*Shu et al.* [25]	⬤	◯	◯	◯
*Our approach*	⬤	⬤	⬤	⬤

## Data Availability

Data are contained within the article.
